# Multigrid Nonlocal Gaussian Mixture Model for Segmentation of Brain Tissues in Magnetic Resonance Images

**DOI:** 10.1155/2016/6727290

**Published:** 2016-08-25

**Authors:** Yunjie Chen, Tianming Zhan, Ji Zhang, Hongyuan Wang

**Affiliations:** ^1^School of Math and Statistics, Nanjing University of Information Science and Technology, Nanjing 210044, China; ^2^Jiangsu Key Laboratory of Meteorological Observation and Information Processing, Nanjing University of Information Science and Technology, Nanjing 210044, China; ^3^School of Information Science and Engineering, Changzhou University, Changzhou 213164, China

## Abstract

We propose a novel segmentation method based on regional and nonlocal information to overcome the impact of image intensity inhomogeneities and noise in human brain magnetic resonance images. With the consideration of the spatial distribution of different tissues in brain images, our method does not need preestimation or precorrection procedures for intensity inhomogeneities and noise. A nonlocal information based Gaussian mixture model (NGMM) is proposed to reduce the effect of noise. To reduce the effect of intensity inhomogeneity, the multigrid nonlocal Gaussian mixture model (MNGMM) is proposed to segment brain MR images in each nonoverlapping multigrid generated by using a new multigrid generation method. Therefore the proposed model can simultaneously overcome the impact of noise and intensity inhomogeneity and automatically classify 2D and 3D MR data into tissues of white matter, gray matter, and cerebral spinal fluid. To maintain the statistical reliability and spatial continuity of the segmentation, a fusion strategy is adopted to integrate the clustering results from different grid. The experiments on synthetic and clinical brain MR images demonstrate the superior performance of the proposed model comparing with several state-of-the-art algorithms.

## 1. Introduction

Magnetic resonance imaging (MRI) is a helpful method for diagnosis of brain diseases, such as Alzheimer's disease and schizophrenia. Accurate tissues segmentation, including gray matter (GM), white matter (WM), and cerebrospinal fluid (CSF), plays an important role in clinical practice and hence has attracted extensive research attention.

Many methods have been proposed for MR image segmentation in the past several decades. These approaches can be classified in terms of different criteria. For example, edge based methods [1, 2], region based methods [3, 4], and clustering based methods [5–7]. Unfortunately, most segmentation methods are hindered by various imaging artifacts such as noise and intensity inhomogeneities.

Intensity inhomogeneity, also known as bias field, arises from the imperfections of the image acquisition process and changes the absolute intensity for a given tissue class in different locations, which usually makes the intensity distribution within a particular tissue class flatter. Most traditional intensity based methods cannot obtain satisfactory results due to the impact of intensity inhomogeneity.

The observed MRI signal *Y* is the product of the true signal *X* generated by the underlying anatomy and a spatially varying field factor *B* and an additive noise *N*:

(1) $$\begin{array}{c} Y_{i}=X_{i}B_{i}+N_{i}\;\forall i\in \left\{1,2,\ldots ,M\right\}, \end{array}$$

where *Y*_*i*_, *X*_*i*_, *B*_*i*_, and *N*_*i*_ are the observed intensity, true intensity, bias field, and noise at the *i*th voxel, respectively. *M* is the total number of pixels in the MR image. Many techniques [6, 8, 9] often ignore the noise and take the logarithmic transform on both sides of (1):

(2) $$\begin{array}{c} \log\left(\;Y\;\right)=\log\left(\;X\;\right)+\log\left(\;B\;\right). \end{array}$$

Many methods have been proposed to correct or estimate intensity inhomogeneities. Collewet et al. proposed a method based on measuring the coil sensitivity functions [8]. Based on the observation that the bias field is smooth, another group of methods overcome the impact of intensity inhomogeneity without estimating the bias field [9, 10]. However, most of them may lose edge information [11].

In this paper, we first propose an improved nonlocal Gaussian mixture model by introducing the nonlocal information into GMM model to reduce the effect of the noise. Then, a new multigrid generation method is presented, and we simplify the NGMM into a local version to eliminate the effect of the bias field and the intensity variation of intratissues. Finally, we propose a fusion strategy to integrate the results from different local regions. The experiments on both synthetic and clinical brain MR images show that our method can obtain more accurate results.

## 2. Nonlocal Gaussian Mixture Model

Gaussian mixture model (GMM) has been widely used in many applications due to its excellent approximation properties. Suppose a MR image has a mixture of *K* components, the mixture density function of pixel *i* can be written as

(3) $$\begin{array}{c} p\left(\;Y_{i}\;|\;\theta \;\right)=\overset{K}{\underset{k=1}{\sum }}\pi_{k}p\left(\;Y_{i}\;|\;\theta_{k}\;\right), \end{array}$$

where *π*_*k*_ is the mixture weights and *θ* = (*θ*_1_, *θ*_2_,…, *θ*_*K*_) is the parameter vector. *p*(*Y*_*i*_∣*θ*_*K*_) is a standard Gaussian distribution of the *k*th component and *θ*_*k*_ = (*μ*_*k*_, *σ*_*k*_) contains the parameters of the Gaussian distribution. *μ*_*k*_ and *σ*_*k*_ are the mean and variance, respectively. Then the entire distribution can be written as

(4) $$\begin{array}{c} p\left(\;Y\;|\;\theta \;\right)=\overset{M}{\underset{i=1}{\prod }}p\left(\;Y_{i}\;|\;\theta \;\right)=L\left(\;\theta \;|\;Y\;\right). \end{array}$$

The problem is how to find the best parameters *θ*:

(5) $$\begin{array}{c} \theta^{*}=\arg\max L\left(\;\theta \;|\;Y\;\right). \end{array}$$

Equation (5) can be calculated by using the expectation-maximization (EM) method [7]. In the E-Step, the algorithm calculates the expected value of the *k*th weight:

(6) pk ∣ Yi,θ=πkpkYi ∣ θ∑j=1KπjpjYi ∣ θ,αk=∑i=1Npk ∣ Yi,θ.

In the M-Step, the parameters of the *k*th Gaussian distribution can be calculated:

(7) πk=1Nαk,μk=∑i=1NYipk ∣ Yi,θ∑i=1Npk ∣ Yi,θ,σk=∑i=1NYi−μk2pk ∣ Yi,θ∑i=1Npk ∣ Yi,θ.

Based on the initialization, *α* and *θ* are calculated iteratively until the stop criteria are reached. Finally, the pixel *i* can be classified into the *k*th class when {*k*∣*p*(*k*∣*Y*_*i*_, *θ*) > *p*(*j*∣*Y*_*i*_, *θ*), *j* ∈ {1,2,…, *K*}  *j* ≠ *k*}. From (3), we can find that the GMM only considers the intensity distribution, which makes the method sensitive to the intensity inhomogeneity and noise.

In order to reduce the effect of the intensity inhomogeneity, Wells et al. [6] proposed a method to simultaneously estimate the bias field and segment the image into different classes. However, the method only addressed the bias field without analyzing the inhomogeneities in inner tissues. In order to ensure the smoothness of the bias field, a low-pass filter is utilized to convolve the bias field, which makes the estimated bias field inaccurate. Furthermore, the method is sensitive to the noise.

In order to reduce the effect of the noise and preserve more detailed information, we improve the Gaussian mixture model by using nonlocal information. The nonlocal information has been widely used for denoising purposes [12, 13]. Following the idea of nonlocal means method [12], we use the nonlocal information to adapt *p*(*k*∣*Y*, *θ*) which can be defined as

(8) NLpk ∣ Yi,θ=∑j=1NWi,jpk ∣ Yj,θ,k=1,…,K,

where *W*(*i*, *j*) is the weight function based on the similarity between the neighbor patch of each neighbor pixel to that of center pixel and satisfies the conditions 0 ≤ *W*(*i*, *j*) ≤ 1 and ∑_*j*=1_^*N*^ *W*(*i*, *j*) = 1. For pixel *i* and its neighbor pixel *j*, the weight function is defined as

(9) $$\begin{array}{c} W\left(\;i,j\;\right)=\frac{e^{-{\left\|\Delta_{i}-\Delta_{j}\right\|}_{2,r}^{2}/h^{2}}}{\sum_{j=1}^{N}e^{-{\left\|\Delta_{i}-\Delta_{j}\right\|}_{2,r}^{2}/h^{2}}}, \end{array}$$

where Δ_*i*_ and Δ_*j*_ are the neighbor patches around pixels *i* and *j* with width 2 × *p* + 1. Broadly speaking, if the neighbor patches of two pixels *i* and *j* are similar, it is more probable that these pixels belong to the same tissue, and the corresponding values of weight function would be higher. Conversely, if these two pixels are quite different, the value of the weight function should be small. *h* acts as a filtering parameter to control the decay of the exponential function. ‖·‖ is the Euclidean distance and *r* is the standard deviation of the Gaussian kernel. Due to the fast decay of the exponential kernel, large distances between estimated patches lead to nearly zero weights. Essentially, the weight function aims to take advantage of the redundancy present in natural structures. Therefore, by using nonlocal information, the nonlocal Gaussian mixture model can reduce the effect of noise and preserve details of the edges.

## 3. Multigrid Nonlocal Gaussian Mixture Method (MGMM)

Without considering the intensity inhomogeneity, the nonlocal Gaussian mixture model can only reduce the effect of noise but cannot obtain satisfactory results for the image containing severe intensity inhomogeneities. Zhu and Jiang [14] proposed a multicontext fuzzy clustering method (MCFC) to reduce the effect of intensity inhomogeneity by using fuzzy clustering method on each nonoverlapping regions and a fusion strategy to integrate the clustering outcomes form different regions. However, this method is sensitive to noise and it only uses traditional multigrid generation method, which makes the method inaccurate. Following the idea of MCFC, we improve the GMM by using the nonoverlapping multigrid. This idea is based on the following four assumptions:

(1) The brain image has been skull-stripped. In this paper, we use the cut based method [15].
(2) Bias field *B* is smooth and slowly varying.
(3) Within each grid, the number of clusters must equal *K* and there are considerable numbers of pixels in each tissue class.
(4) Within a grid, all pixels of the same tissue have similar true intensities.

**Figure 1:**
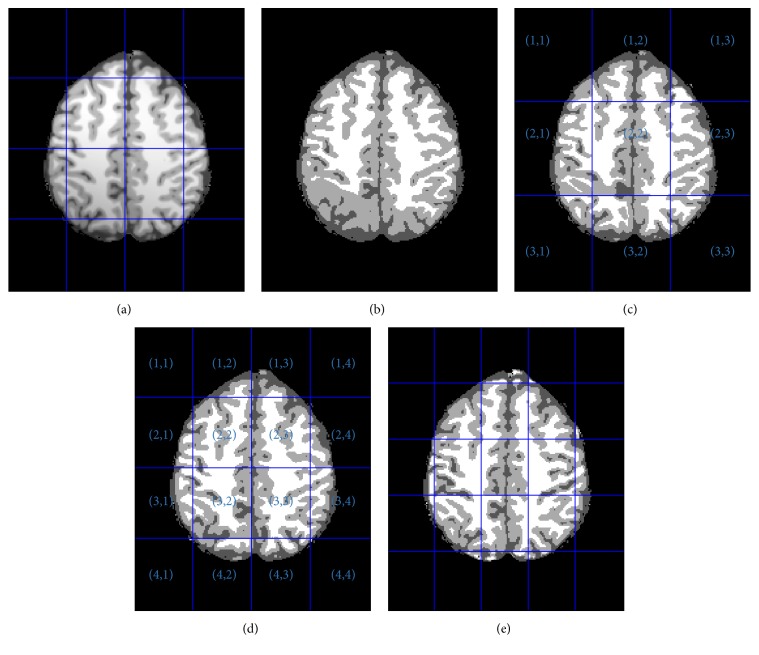
Brain image segmentation: (a) original T1-weighted image; (b) GMM segmentation; (c)–(e) MGMM segmentation.

The brain image without skull only has cerebrospinal fluid, gray matter, and white matter. Then, we set *K* = 3 with assumption (3). The bias field is smooth and slowly varying, which makes it probable that the bias field values in small local region be regarded as constant. Then the multigrid segmentation method is less sensitive to the bias field. However, each pixel is processed only in its single local grid, which makes the method very sensitive to the size of the grid and unable to preserve the statistical reliability and spatial continuity of segmentation results. This can be illustrated in Figure 1.

Figure 1 shows the segmentation results of GMM and MGMM applied on a synthetic 3 T MR image, which were created by using the MRI simulator (Brain Imaging Center at the Montreal Neurological Institute, McGill University, Montreal). There are many advantages for using these synthetic images rather than real image data volumes for validating segmentation methods. The simulator can provide full three-dimensional data volumes which have been simulated using three sequences (T1-, T2-, and PD-weighted) and a variety of slice thicknesses, noise levels, and intensity inhomogeneity levels, providing the ground truth of the image data. In this paper, the parameters of the simulated data sets are as follows: Phantom: normal; slice thickness: 1 mm; scan technique: SFLASH; TR = 18 msec; flip angle = 30 degrees; TE = 10 msec. The dimension of the image data sets is 181 × 217 × 181. The parameters of Figure 1(a) are as follows: noise level 0% and INU (RF) level 100%. Figure 1(a) shows the initial image, which is uniformly divided into 4 × 4 nonoverlapping grids.  Figure 1(b) shows the results of GMM. Due to the effect of the bias field, some voxels of WM and CSF have been misclassified into GM. Figures 1(c)–1(e) are segmentation results of MGMM when the image is divided into 3 × 3, 4 × 4, and 5 × 5 grids, respectively.

It can be seen from the grids (2, 2) and (3, 2) in Figure 1(c) or from the grids (3, 2) and (4, 2) in Figure 1(d) that the variation of intensity distributions of neighbor patches would, more or less, lead to discontinuities across the grids' boundaries. Furthermore, when the bias field is severe, grids with large size can satisfy assumption (3); however, this would make the method sensitive to the bias field and does not satisfy assumption (4). It is hard to hold assumption (3) in some grids when the size of the grids is small. For example, there are no WM pixels in the grid (3, 1) of Figure 1(e), which makes some pixels of GM misclassified into WM. It can also be found in grid (3, 2) of Figure 1(e) that tissues of WM and GM predominate the grid, which yields a deviant intensity distribution far from that typical of the brain. The GMM misclassifies some GM pixels with relatively low intensity into the class of CSF.

### 3.1. New Multigrid Generation Method

**Figure 2:**
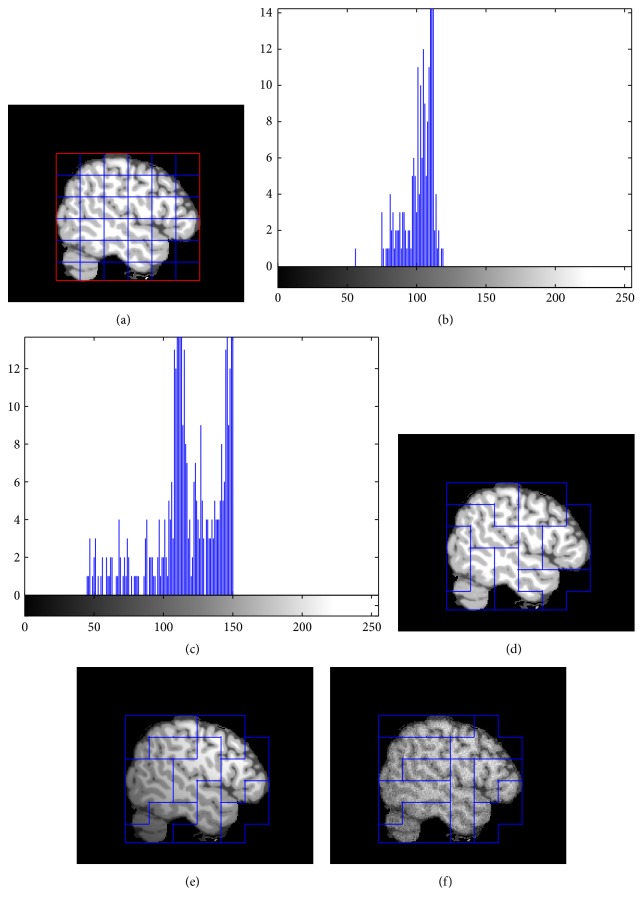
Generation of the grid: (a) initial results; (b) histogram of patch (6,2); (c) histogram of patch (3, 3); (d)–(f) results of multigrid.

In this paper we present a new method to generate the multigrid. Firstly, the boundary of the brain needs to be found, because there are a large number of pixels belonging to the background in brain MR images, which usually affect the accuracy of segmentation methods. Secondly, the brain region is divided into *N* × *N* small nonoverlapping grids. The generated nonoverlapping grids may not satisfy assumption (3).  Figure 2 shows the generated multigrid on brain MR images. In this paper, we set *N* = 6. It can be seen from Figure 2(a) that the grids (1, 1) and (6, 3) only contain some CSF pixels and the grids (1, 6) and (6, 6) have no brain tissues. Furthermore, the grid (6, 1) has no pixels of the WM. The NGMM cannot obtain accurate results based on these grids. In order to deal with this problem, the small grids need to be combined. The combine process includes 6 steps as follows.

**Step 1 .**

Define a matrix *L* with the same size of the multigrid and a variable *N*_number_ = 1 to count the serial number of the final patches.

**Step 2 .**

Classify every grid into *K* classes.

**Step 3 .**

Find the worst grid (*x*, *y*) and set *L*(*x*, *y*) = *N*_number_.

**Step 4 .**

Find the preferred grid (*x*′, *y*′) to be combined into the patch {(*x*, *y*)∣*L*(*x*, *y*) = *N*_number_} and set *L*(*x*′, *y*′) = *N*_number_. Repeat this process *N*_search_ times.

**Step 5 .**

Set *N*_number_ = *N*_number_ + 1 and go to Step 3 until all grids have been labeled except those nonbrain grids.

**Step 6 .**

Combine single patches into the preferred patches nearby.

In Step 1, matrix *L* is defined to sign the multigrid and initialized to zero for ∀*x*, *y*. The variable *N*_number_ is used to count the serial numbers of the final patches and initialized as 1. In Step 2, every grid is classified into *K* classes by using Fuzzy Clustering Means (FCM) method, which can classify grids efficiently. We assume that assumption (3) holds in all grids and all patches are classified into 3 classes except those background grids. The histograms of the grids (6,2) and (3, 3) are shown in Figures 2(b) and 2(c), respectively. There are only GM and some CSF pixels in the grid (6,2), which make the distribution of the pixels' intensities more compact than that of (3, 3).

In Step 3, we first calculate an inner distance for every grid. The inner distance is defined as min(|*C*_CSF_ − *C*_GM_|, |*C*_WM_ − *C*_GM_|), where *C*_CSF_, *C*_GM_, and *C*_WM_ are the intensity centers of CSF, GM, and WM, respectively. The worst grid (*x*, *y*) with the smallest inner distance can be found easily and *L*(*x*, *y*) is set as *N*_number_. In Step 4, the grids $\left\{(\hat{x},\hat{y})|L(\hat{x},\hat{y})=0\right\}$, which are next to the patch {(*x*, *y*)∣*L*(*x*, *y*) = *N*_number_} need to be found. Then, every grid $\left(\hat{x},\hat{y}\right)$ is combined into the patch {(*x*, *y*)∣*L*(*x*, *y*) = *N*_number_} independently and the inner distance of the corresponding combined patch is calculated. The grid with the largest inner distance is regarded as the preferred grid to be combined into patch {(*x*, *y*)∣*L*(*x*, *y*) = *N*_number_}. This process should be repeated for *N*_search_ times, where *N*_search_ satisfies *N*_search_ *∗N*_grid_ ≤ *αN*_brain_. *N*_grid_ is the size of the grids. *N*_brain_ is the amount of tissue pixels in the input image. *α* is the control parameter. It is hard to hold assumption (4) with large *α*. In this paper, we set *α* = 0.1, then *N*_search_ = 3. Furthermore the width and height of every patch are no more than 1/2 of the brain region's width and height.

After Step 5, all grids have been labeled; however, there may be some grids that have not been combined with any neighbor patch. These grids are set as single grids. It is hard to hold assumption (3) in these grids. In order to deal with this problem, we use similar strategy shown in Step 4 to combine the single grids into best neighbor patch. Firstly, we find one of the single grids. Secondly, we analyze the neighbor patches, which are next to the single grid, to find the patch with largest inner distance as the preferred patch. The single grid is combined into the preferred patch. The process is repeated until all single grids have been combined into their preferred patch.

Figures 2(d)–2(f) show the results of the new multigrid generation method on three synthetic brain MR images, with the following parameters: noise levels are 0%, 0%, and 5%, respectively, and INU (RF) levels are 0%, 100%, and 40%, respectively. It can be seen that our method can generate good multigrid even when the image has strong noise or bias field.

### 3.2. Multigrid Nonlocal Gaussian Mixture Model (MNGMM)

Assumption (3) can be retained by using the improved multigrid. However, due to the effect of the bias field, the variation of intensity distributions of neighbor patches would, more or less, lead to boundaries discontinuous across edges of some grids. Despite this problem, the assumptions for simplifying the image model are still correct in most cases, due to the complicated and convoluted structure of human brain as mentioned above. In other words, most grids would yield good judgments based on the expected values *p*(*k*∣*Y*_*i*_, *θ*). A very natural idea is that the adverse impact of some grids could be overcome by all the good clustering results from other neighbor patches. Such a consideration leads to the development of a novel method called the multigrid nonlocal Gaussian mixture model (MNGMM) that can take advantage of the adaptation of local clustering but also keep the classifications spatially continuous and statistically reliable.

MNGMM includes two basic parts: clustering and information fusion. The rationale of MNGMM can be described as follows. First, all grids need to be classified by using NGMM to obtain the corresponding expected values *p*(*k*∣*Y*_*i*_, *θ*). For every pixel *i*, it is easy to find the corresponding grid and obtain its expected values *p*(*k*∣*Y*_*i*_, *θ*) in the grid. The distribution information of the neighbor grids *Q*_*ij*_, *j* = 1,…, *N*_*i*_ next to *i* is used to calculate *p*(*k*∣*Y*_*i*_, *θ*_*j*_), where *θ*_*j*_ is the distribution parameter of *Q*_*ij*_ and *N*_*i*_ is the total number of the neighbor grids. In the information fusion stage, all these expected values are integrated with a strategy *F* to adapt the current expected values. Consequently, for each pixel *P*, MNGMM can be summarized as follows.

**Step 1 .**

One has

(10) $$\begin{array}{c} Q_{ij}=\left\{\;p\left(\;k\;|\;Y_{i},\theta_{j}\;\right)\;\right\},\;k=1,\ldots ,K;\;\;j=1,\ldots ,N_{i}. \end{array}$$

**Step 2 .**

One has

(11) pk ∣ Yi,θ=Fpk ∣ Y,θ,pk ∣ Y,θ1,…,pk ∣ Y,θNi.

Here, we name *p*(*k*∣*Y*_*i*_, *θ*) as *p*_*k*,0_ and *p*(*k*∣*Y*_*i*_, *θ*_*j*_), *j* = 1,…, *N*_*i*_ as *p*_*k*,*j*_; the strategy is

(12) $$\begin{array}{c} F\left(\;p_{k,0},p_{k,1},\ldots ,p_{k,N_{i}}\;\right)=\overset{N_{i}}{\underset{j=0}{\sum }}\frac{p\left(Y_{i}\;|\;\theta_{k,j}\right)}{\sum_{l=0}^{N_{i}}p\left(Y_{i}\;|\;\theta_{k,l}\right)}p_{k,j}. \end{array}$$

In our method, only pixels on edge regions of the grid may be misclassified into other class. The intensities of the pixels are continuous across the edges of the neighbor grids, which makes it probable that the expected values of the misclassified pixels be weighted averaged by using the information of the neighbor grids. As a result, the weighted averaging values of different grids make the final result more reliable. Moreover, the weighted averaging operation can also preserve spatial continuity of the membership distributions of each tissue class. Finally, maximum expected values *p*(*k*∣*Y*_*i*_, *θ*) principle is used to obtain the segmentation results from the weighted averaging values.

## 4. Results

**Figure 3:**
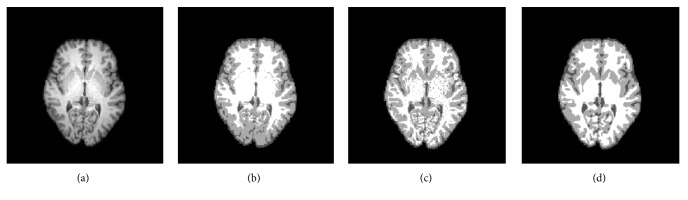
Segmentation results for a clinical 3 T MR image: (a) original image; (b) segmentation result of GMM; (c) segmentation result of Wells; (d) segmentation result of MNGMM.

In order to show the robustness of our method, we compared our method with GMM and Wells method on a clinical 3 T MR image, which has severe intensity inhomogeneity and noise. The acquisition parameters of the real data are as follows: slice thickness 1 mm, echo delay time (TE) 35 msec, repetition time (TR) 450 msec, and flip angle 90 deg. The size of the data is 256 × 256 × 170. From the image, we can find that most intensities of putamen are higher than those of the cortex, which also belong to the GM. GMM misclassifies putamen into WM since some intensities of putamen are closer to WM than to GM, which can be shown in Figure 3(b). The result of the Wells method [6] is shown in Figure 3(c). It can be seen from the result that the method can reduce the effect of the bias field; however, with the effect of the inhomogeneities in inner tissues, some pixels of putamen have been misclassified into WM and the method is sensitive to the noise. In contrast, MNGMM can yield satisfactory results, which are more compatible with human visual perception.

**Figure 4:**
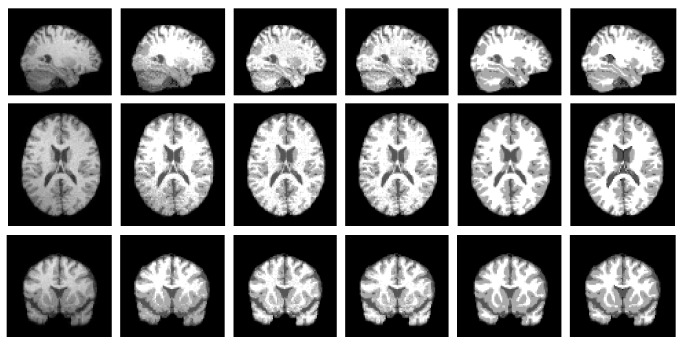
Segmentation results of synthetic data with the parameter noise level 5% and INU level 80%. The left column shows the initial image. The second column to the fifth column show the results of GMM, Wells, MCFC, and MNGMM, respectively. The right column is the ground truth.

In order to quantitatively show the performances of the proposed model, we compared our method with GMM, Wells method, MCFC, and MNGMM on the synthetic data from MRI simulator with the following parameters: noise level 5% and INU level 80%. The results are shown in Figure 4. The left column shows the initial image. The end column shows the ground truth. The second column to the fifth column show the segment results of GMM, Wells, MCFC, and MNGMM, respectively. Due to the effect of the intensity inhomogeneity, many pixels of WM have been misclassified into GM in the result of GMM. Wells and MCFC can reduce the effect of the intensity inhomogeneities; however, they are affected by the noise. By using the nonlocal information and multigrid information, MNGMM can yield much more complete and continuous results, which are very similar to the ground truth.

**Figure 5:**
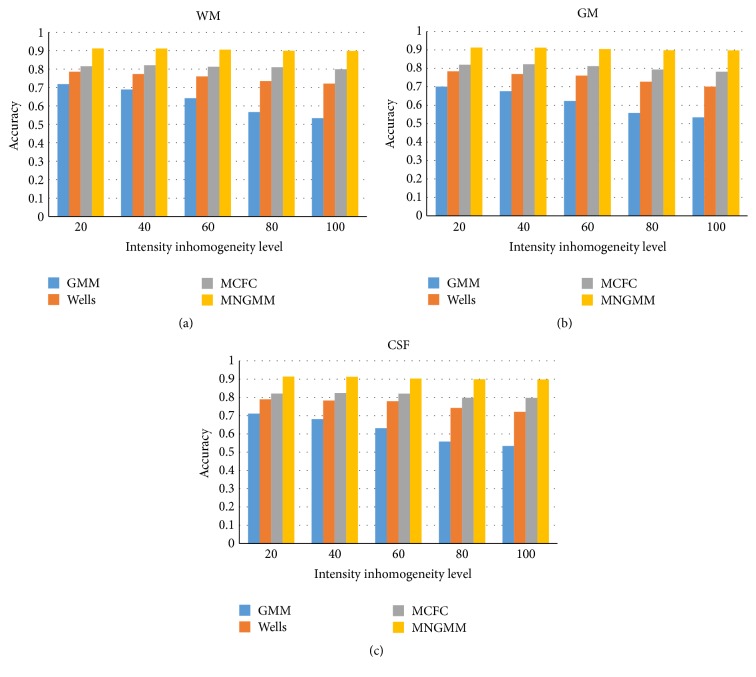
Average JS values of the segmentation results of WM (a), GM (b), and CSF (c) obtained by applying five segmentation methods to simulated brain MR images with increasing levels of intensity inhomogeneity.

We use Jaccard similarity value (JS) [6] to quantitatively evaluate the performance of a classification method. The value of JS ranges from 0 to 1, with a higher value representing a more accurate segmentation result. The statistical results (means and standard deviations of JS values for WM, GM, and CSF) are listed in Table 1. The results demonstrate that our method produces the most accurate results and has the best ability and robustness to the noisy images (with lower standard deviations of JS values and higher mean of JS values when the noise increases), especially in the area with abundant textures (with higher JS values for CSF tissue). We also apply the above five methods to the segmentation of 40 whole synthetic MR image data sets, in which the level of intensity inhomogeneity ranges from 20% to 100%. The segmentation accuracy is measured in terms of the average JS of WM, GM, and CSF delineation and is shown in Figure 5. Both visual and quantitative comparisons show that our method is more robust to the intensity inhomogeneity and can obtain more accurate results.

**Figure 6:**
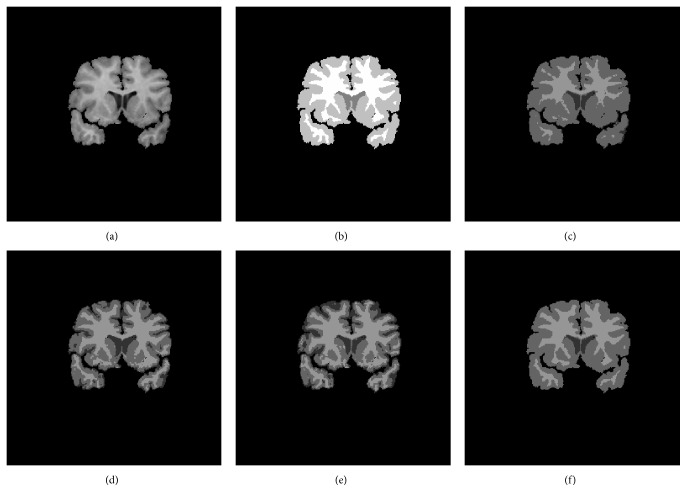
Segmentation results of real data from IBSR with the name 12_3 (39th image). (a) Initial image, (b) ground truth, and (c–f) the segmentation results of GMM, Wells, MCFC, and MNGMM.

Figure 6 shows the segmentation results on a real brain MR data from the Internet brain segmentation repository (IBSR at http://www.cma.mgh.harvard.edu/ibsr/) with the name 12_3 (39th image). The intensity distribution of the basal ganglia is midway between the assumed distributions of GM and WM and the basal ganglia have low contrast. From the results, we can find that our method can obtain accurate result. In order to quantitatively evaluate the benefits, we segmented 20 standard sets of real brain MR data from IBSR by using GMM, Wells method, MCFC, and MNGMM. The average quantitative results of GM, WM, and CSF are listed in Table 2. It can be seen that our method is more accurate than others.

## 5. Discussion

**Figure 7:**
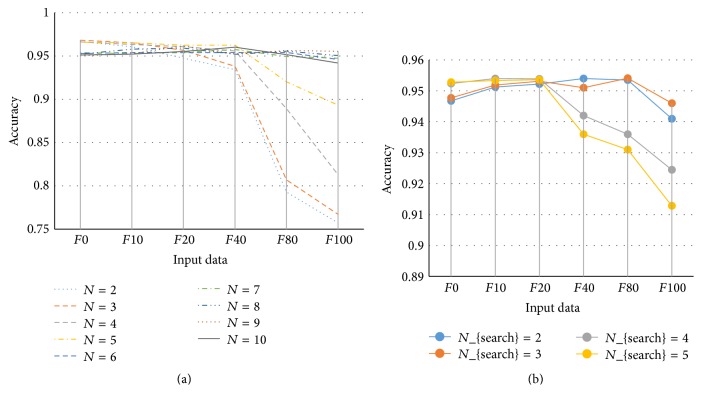
Accuracies of the segmentation with different *N* and *N*_search_.

In our work, the number of grids *N* is set as a constant. The choices of the divided number should be based on the size of the brain region and intensity inhomogeneity level. A smaller divided number will make the brain region only divided into few patches, which makes the proposed method sensitive to intensity inhomogeneity. A larger divided number will make the method less efficient. We have studied the relationship between size of patches and segmentation accuracies.  Figure 7 shows the accuracies of the segmentation to simulated images with different parameters. In fact, the larger the patch, the more the data to be clustered; the greater the similarity between the intensity distribution of the patch and that of the input image is, the more reliable the clustering results are. However, assumptions 2 and 4 require smaller patches. The left column of Figure 6 shows the accuracies of the results using different *N* when generating the multigrid on the input data with the following parameters: noise level, 0%, and intensity inhomogeneity level, 0%, 10%, 20%, 40%, 80%, and 100%, respectively. It can be seen that with the increase of intensity inhomogeneities the accuracies decrease when *N* is small, which also means that the patch is bigger. At this time, assumptions (2) and (4) cannot be satisfied and the accuracies decrease very quickly. When *N* = 6, 7, 8, 9 the results have similar accuracies. We also analyzed the influence of *N*_search_. The right column of Figure 7 shows the accuracies with different *N*_search_ when *N* = 6. It can be seen from the results that the accuracy is decreasing with the increase of *N*_search_ when the intensity inhomogeneities increase. This is because the bigger *N*_search_ leads to bigger patches, which means that assumptions (2) and (4) cannot hold.

## 6. Conclusions

In this paper, we have presented a theoretically simple approach to automatically segment 2D or 3D human brain MRI data. To reduce the effect of intensity inhomogeneity, a multigrid Gaussian mixture model has been proposed. In order to reduce the effect of noise, we improve the Gaussian mixture by using the nonlocal information, which can preserve more detailed information. Experimental results have proved that our method outperforms other segmentation methods when segmenting images with severe intensity inhomogeneities and noise.

## Figures and Tables

**Table 1:** The average JS values (mean ± standard deviation) of GM, WM, and CSF segmentation obtained by applying four algorithms to T1-weighted brain MR images with increasing level of noise.

Algorithm	Tissues	3%	5%	7%	9%
GMM	WM	0.8512 ± 0.051	0.7532 ± 0.047	0.6574 ± 0.064	0.6135 ± 0.067
	GM	0.8478 ± 0.059	0.7231 ± 0.065	0.6326 ± 0.046	0.6012 ± 0.056
	CSF	0.8547 ± 0.048	0.7447 ± 0.054	0.6236 ± 0.043	0.6103 ± 0.055

Wells	WM	0.9201 ± 0.071	0.7932 ± 0.069	0.7154 ± 0.061	0.6843 ± 0.062
	GM	0.9102 ± 0.056	0.7863 ± 0.048	0.7001 ± 0.044	0.6632 ± 0.051
	CSF	0.8842 ± 0.052	0.7731 ± 0.059	0.7011 ± 0.061	0.6691 ± 0.058

MCFC	WM	0.9382 ± 0.051	0.8131 ± 0.053	0.7320 ± 0.046	0.6914 ± 0.038
	GM	0.9262 ± 0.048	0.7914 ± 0.046	0.7250 ± 0.047	0.6853 ± 0.032
	CSF	0.8937 ± 0.046	0.7724 ± 0.055	0.7123 ± 0.059	0.6749 ± 0.054

MNGMM	WM	0.9328 ± 0.019	0.9257 ± 0.032	0.9231 ± 0.031	0.9105 ± 0.032
	GM	0.9331 ± 0.017	0.9216 ± 0.038	0.9187 ± 0.034	0.9073 ± 0.038
	CSF	0.9293 ± 0.022	0.9211 ± 0.039	0.9127 ± 0.028	0.9005 ± 0.041

**Table 2:** The average JS values for the real MR images segmentation (%).

	GMM	Wells	MCFC	MNGMM
JS of WM	61.32	72.13	80.87	90.31
JS of GM	59.23	71.35	78.31	88.37
JS of CSF	57.87	78.32	76.45	89.52
